# Successful surgical removal of a cutaneous lesion compatible with gigantic vascular eccrine spiradenoma: a rare case report

**DOI:** 10.1093/jscr/rjad259

**Published:** 2023-06-05

**Authors:** Reem A Alnafee, Aram K Almasaud, Abdullah M Alshamrani, Saud D Alrasheedi, Abdulrahman J Alzahrani, Adel S Alqahtani

**Affiliations:** General Surgery Department, Security Forces Hospital Program, Riyadh 11481, Saudi Arabia; General Surgery Department, Security Forces Hospital Program, Riyadh 11481, Saudi Arabia; General Surgery Department, Security Forces Hospital Program, Riyadh 11481, Saudi Arabia; General Surgery Department, Security Forces Hospital Program, Riyadh 11481, Saudi Arabia; General Surgery Department, Security Forces Hospital Program, Riyadh 11481, Saudi Arabia; General Surgery Department, Security Forces Hospital Program, Riyadh 11481, Saudi Arabia

## Abstract

Giant vascular eccrine spiradenoma (GVES) is an uncommon type of eccrine spiradenoma (ES). Compared to an ES, this is characterized by a greater degree of vascularity and a bigger size overall. In clinical practice, it is frequently mistaken for a vascular or malignant tumor. To achieve an accurate diagnosis of GVES via biopsy and successfully perform the surgical removal of a cutaneous lesion in the left upper abdomen compatible with GVES. We present a 61-year-old female with the lesion accompanied by on-and-off pain, bloody discharge and skin changes surrounding the mass treated surgically. However, there was no fever, weight loss, trauma or family history of malignancy or cancer managed by surgical excision. The patient recovered well postoperatively and was discharged on the same day with a 2-week follow-up. The wound got healed, the clips were removed on Day 7 postoperatively, and there was no requirement for further follow-up.

## INTRODUCTION

Eccrine spiradenoma (ES) is a rare benign cutaneous adnexal tumor from sweat glands [1]. It usually appears as a dermal or subcutaneous lump or papule in the head, neck, trunk or extremities and can cause discomfort and tenderness [2]. Due to its florid vascularity and hemorrhagic characteristics, giant vascular eccrine spiradenoma (GVES) may be misdiagnosed as a vascular lesion or malignant tumor. The macroscopic appearance of a gigantic vascular ES is not distinct. Consequently, this makes clinical identification challenging. Therefore, a biopsy is needed for a precise diagnosis [3].

Cotton *et al.* 1986 first documented two large ESs with significant vascularity in each case [4]. Following the initial two reported cases, 10 further examples of GVES have been published in scientific journals [3, 5]. Most documented cases have been in individuals over 49; however, one case has been reported in males aged 41 [6] and 31 [7]. There is no discernible difference between the rates that affect men and women. In terms of duration, the shortest lasted 3–4 months, while the longest lasted 30 years [8]. According to the research, the lesions’ sizes might range from 2 cm to 7 cm. The list of reported sites includes the head, trunk and limbs. However, no instances of GVES in the upper left abdomen region have been observed.

These tumors’ surfaces were of various colors, including red, gray, purple, blue and pink. The surrounding skin may be smooth, ulcerated or bleeding, depending on the type of tumor. As far as the composition and differentiation of GVES are concerned, there is a widespread consensus that tumors comprise two distinct kinds of cells: giant pale cells in the tumor’s core, while the small basaloid cells can be found on the tumor’s periphery [9, 10]. However, some researchers have come to different results; for example, Ko *et al*. [11] and Georgesen *et al*. [12] documented the immunohistochemical characteristics of GVES and came to the same findings. They discovered that a GVES is composed of three different types of cells: epithelial cells, tiny basal cells and myoepithelial cells.

The GVES is distinguished from other types of spiradenoma by its unusually high degree of vascularization. There are now two hypotheses that can account for the prominent vascularity. The first is that a vascular area of the normal sweat gland gives birth to GVES [4, 12]. Several reports concluded that the tumor was of vascular origin based on discovering an afferent artery of the tumor that drained into the basilic vein revealed by magnetic resonance imaging [13]. According to a different viewpoint [14], the vascular component may have been formed due to degradation in the tumor stroma throughout the tumor’s expansion and maturation. Previous research indicates that the progression of GVES can range from 3 months to 30 years; nonetheless, there was considerable vascularization in each patient. ESs, the most prevalent kind, survived for 15–30 years. However, there was no evident vascularization observed [15–17]. Because of this, it is abundantly evident that the tumor’s vascularization does not directly affect the disease’s duration.

The differential diagnosis of GVES is critical, and many clinical diagnoses, such as chronic expanding hematoma [8], calcifying epithelioma [18], angiolipoma or neuroma, desmoid [19], angiomatous lesion or thrombosis [20], angiosarcoma [4], etc., could be erroneously made. Although malignant ESs have been documented in the past [21, 22], there has not been a single instance of a malignant GVES published in the literature to this day. Hence, we present a case report of a GVES found in an old female’s abdomen, making a differential diagnosis of skin lesions and effective surgical removal.

## CASE REPORT

**Figures 1:**
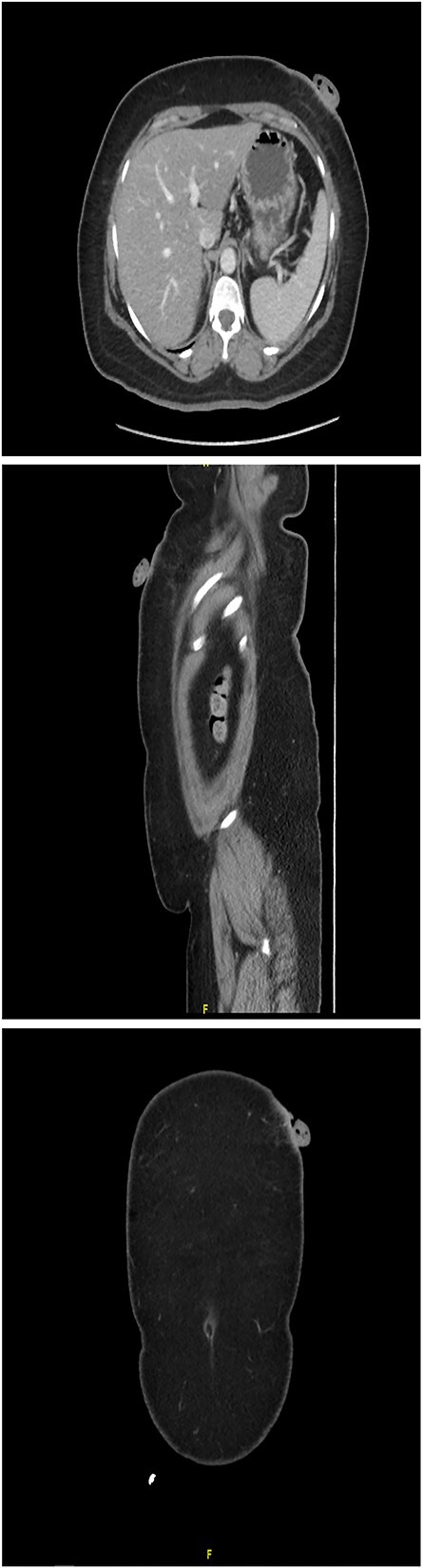
CT scanned abdomen and pelvis showing an anterior left upper abdominal wall skin-based, pedunculated, enhancing soft tissue nodule with a large stalk

A 61-year-old woman with type 2 diabetes, hypertension and dyslipidemia presented to the authors’ hospital’s outpatient department with a left upper abdomen wall tumor that began as a tiny nodule and gradually enlarged over 15 years. The lesion caused pain, bloody leakage and skin changes around the mass. The patient denied having ever had a fever, weight loss, trauma or any family history of cancer. The patient was conscious, alert and not in pain during the physical examination. Local inspection revealed a brown mobile single mass in the left upper abdomen, shaped like a pedunculated polypoid measuring roughly 3 cm × 2 cm with an irregular border. An anterior left upper abdominal wall skin-based, pedunculated, enhancing soft tissue nodule with a giant stalk was seen on computed tomography (CT) scanned abdomen and pelvis (Fig. 1). The nodule measures 1.8 cm × 3.6 cm × 3.6 cm and has no free soft tissue invasion. Based on these observations, surgical excision and histological examination were carried out.

**Figure 2:**
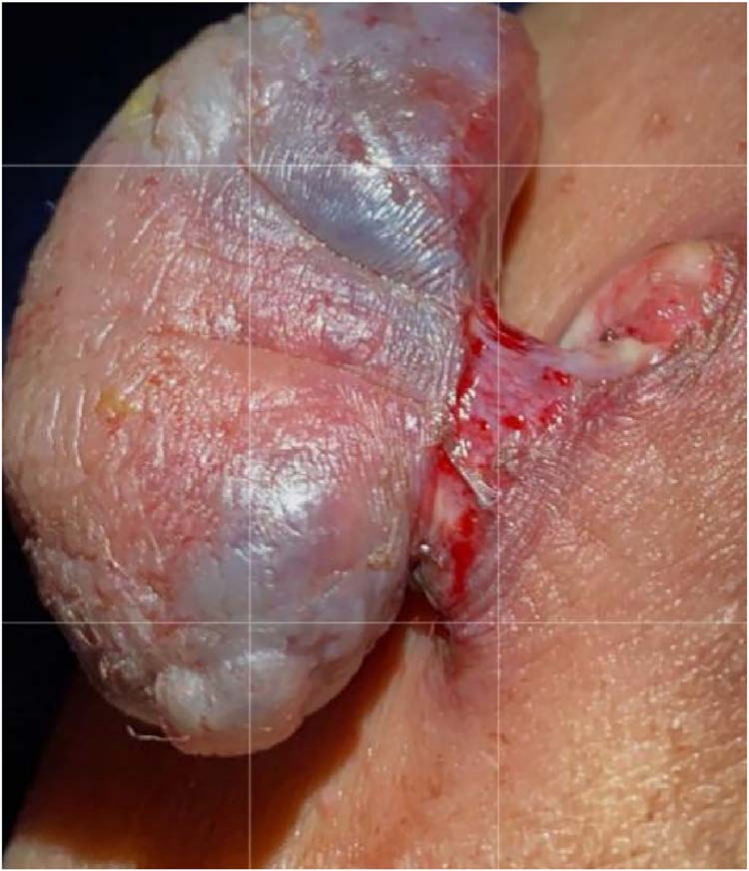
Macroscopic examination: the specimen received in formalin labeled with the patient’s name, MN, as ‘infected skin lesion, left upper abdomen.’ It consisted of skin measuring 3.0 cm × 1.2 cm overlying fibro-fatty tissue measuring 3.0 cm × 2.0 cm × 1.0 cm with polypoid skin lesion measuring 3.5 cm × 2.0 cm × 1.0 cm. The lesion is 1.2 cm away from the deep margin and 0.5 cm from the radial margin

**Figure 3:**
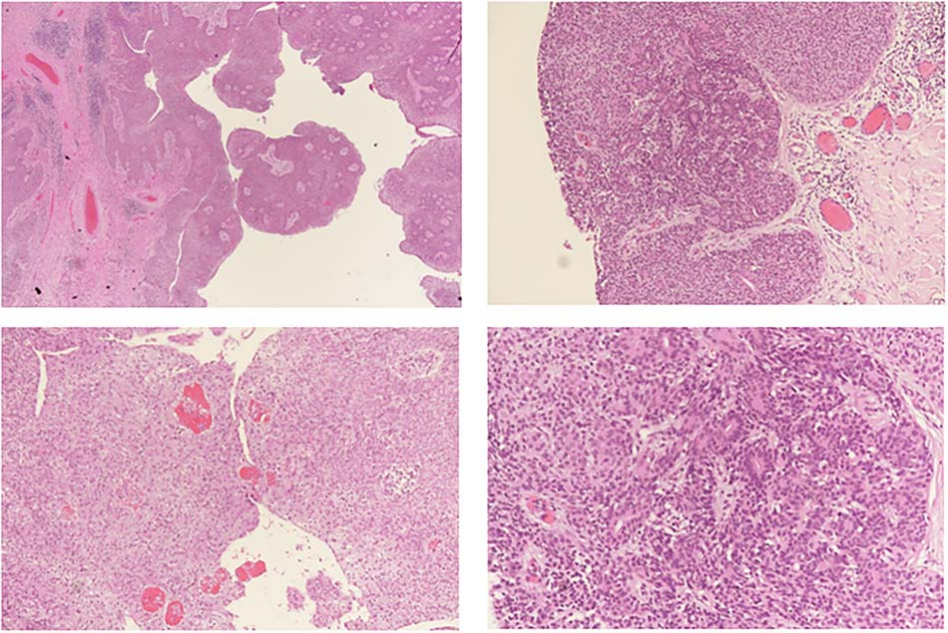
Histopathological analysis: a benign hemorrhagic neoplasm in the dermis with cystic change (**a**). The tumor is composed of two cell populations; small basaloid cells (**b**) and large pale cells (**c**) arranged in a solid fashion with tubular structure formation and numerous intra-lesional vessels (**d**) containing red blood

The surgical approach entailed identifying the lesion, then making an elliptical incision around 5 cm under general anesthesia, resecting the tumor bed with electrocautery, attaining hemostasis, sealing the skin with clips and submitting the specimen for histological evaluation (Fig. 2). A GVES was histologically diagnosed as a cutaneous lesion in the upper left abdomen. The lesion was removed entirely, and the case was investigated by an anatomic pathology consultant, who concurred with the diagnosis (Fig. 3). The patient was assessed and found to be doing well postoperatively. She was discharged on the same day with a 2-week follow-up appointment. On postoperative Day 7, her incision was cleaned and dried, and the clips were removed. The patient was advised of her diagnosis and that no further follow-up was required.

## DISCUSSION

Lauret *et al.* [23] coined ‘giant eccrine spiradenoma’ in 1977. Tumors as big as 12 cm in size have been reported previously. Cotton *et al*. identified GVES, an uncommon variety of ES, in 1986 [4]. They described two examples of massive ES with significant vascularity. The enormous vascular variety differs from ES in size (diameter > 2 cm) and vascularity. As a result of the vascularity and hemorrhagic features, there are increased chances of inaccurate diagnoses by physicians and pathologists. Most described GVES were deep dermal or subcutaneous solitary tumors with a red, gray, purple, blue or pink appearance. The skin on top of the tumor may be smooth, ulcerated or bleeding. Yamakoshi *et al*. [24] described a patient with a GVES on his shoulder with insufficient development space, resulting in a giant pedunculated mass.

Despite previous cases of malignant ESs [21, 22], no malignant GVES has been documented in the literature. A GVES with an uneven infiltrative growth pattern into the surrounding pseudocapsule has been reported previously [12]. Despite the architectural atypia, they classified their case as benign due to the low proliferative index of Ki-67 staining and the absence of mitotic figures. In addition, they stated that the patient would be thoroughly monitored in the future. We proceeded over our case as follows: Age: Our patient was elderly, over 60. Except for one instance on the scalp [25], all GVES cases [4, 12, 21, 22, 24] occurred on the boot or limbs, and our case developed in the abdomen. The lesions appeared as a well-defined, isolated mass ˃2 cm in diameter, with spontaneous bleeding and the potential for discomfort.

She had no visible incisions from the treatment after a 7-day follow-up. It is crucial to examine GVES when evaluating subcutaneous lesions. Surgical excision with clear margins is the therapeutic principle for GVES. Accurate identification of GVES might be difficult for a surgical pathologist, resulting in an incorrect diagnosis. As a result, a high index of suspicion should be maintained, and an alternate differential of a spiradenoma should be explored, particularly in cutaneous neoplasms with extensive vascularity that present clinically as varix or hematomas. Postoperative monitoring is critical for detecting and the treatment of any recurrences.

## CONCLUSION

To conclude, we addressed the case of a massive vascular ES discovered in the abdomen. This fact must be considered while making a differential diagnosis of skin lesions.

## CONFLICT OF INTEREST STATEMENT

None declared.

## FUNDING

None.
